# Secure Smart Cameras by Aggregate-Signcryption with Decryption Fairness for Multi-Receiver IoT Applications

**DOI:** 10.3390/s19020327

**Published:** 2019-01-15

**Authors:** Subhan Ullah, Lucio Marcenaro, Bernhard Rinner

**Affiliations:** 1 Institute of Networked and Embedded Systems, Alpen-Adria-Universität Klagenfurt, Universitätsstraße 65-67, 9020 Klagenfurt, Austria; bernhard.rinner@aau.at; 2 Department of Electrical, Electronic, Telecommunications Engineering and Naval Architecture, University of Genova, Via all’Opera Pia 11, 16145 Genova, Italy; lucio.marcenaro@unige.it

**Keywords:** smart cameras, data security, elliptic-curve signcryption, multi-receiver aggregate-signcryption, Internet of Things, resource efficiency

## Abstract

Smart cameras are key sensors in Internet of Things (IoT) applications and often capture highly sensitive information. Therefore, security and privacy protection is a key concern. This paper introduces a lightweight security approach for smart camera IoT applications based on elliptic-curve (EC) signcryption that performs data signing and encryption in a single step. We deploy signcryption to efficiently protect sensitive data onboard the cameras and secure the data transfer from multiple cameras to multiple monitoring devices. Our multi-sender/multi-receiver approach provides integrity, authenticity, and confidentiality of data with decryption fairness for multiple receivers throughout the entire lifetime of the data. It further provides public verifiability and forward secrecy of data. Our certificateless multi-receiver aggregate-signcryption protection has been implemented for a smart camera IoT scenario, and the runtime and communication effort has been compared with single-sender/single-receiver and multi-sender/single-receiver setups.

## 1. Introduction

Smart cameras are real-time computer vision systems that combine sensing, processing, and communication capabilities on an embedded device [1]. These devices have undergone tremendous advances over the last decade and play an important role in several Internet of Things (IoT) applications [2,3,4]. Smart cameras may capture sensitive data and reveal the identities as well as clues about the habits, preferences, and social interests of the captured persons [5]. Thus, security and privacy has become a major concern due to their widespread deployment, the sensitive nature of the captured data and the open infrastructure [6]. The effectiveness and efficiency of the protection techniques is a particular challenge due to the resource limitations of the smart-camera devices.

This paper addresses this challenge and introduces a lightweight security approach for smart camera IoT applications. Our approach is based on EC-based signcryption which performs data signing and encryption in a single step [7]. In our previous work, we deployed individual- and aggregate-signcryption for protecting sensitive data onboard the cameras and secure the transfer from a single camera or clusters of cameras to a single monitoring device [8,9]. In this paper, we generalize this approach to efficiently protect the data from multiple cameras to multiple monitoring devices. This multi-sender/multi-receiver approach provides integrity, authenticity and confidentiality of data with decryption fairness for multi-receiver throughout the entire lifetime of the data. It further provides public verifiability (i.e., any trusted party can verify the authenticity) and forward secrecy (i.e., the confidentiality of incoming data is not compromised, if someone has compromised the current or past session keys) of data.

The contribution of this paper includes the deployment of EC-based aggregate-signcryption for multi-receiver in an IoT scenario. We adopt a certificateless approach by using a key generation center (KGC) and avoid the key escrow [10] problem of key sharing and authentication. This certificateless multi-receiver aggregate-signcryption approach has been implemented and evaluated in a smart-camera IoT scenario. The proposed approach saves 32.89% and 28.90% of computational time as compared to individual- and aggregate-signcryption in a multi-sender/multi-receiver scenario.

The rest of the paper is organized as follows: Section 2 discusses the state-of-the-art. Section 3 introduces the system architecture, assumptions, and threat model. Section 4 and Section 5 describe the proposed solution and experimental evaluation, respectively. Finally, Section 6 concludes the paper.

## 2. State-of-the-Art

In the following subsections, we discuss first the protection of data onboard smart cameras, second the protection within a network of smart cameras and third the use-cases of smart cameras. Finally, we discuss the efficiency of the proposed signcryption technique and provide a comparison with other security approaches.

### 2.1. Onboard Protection on Smart Cameras

A smart camera [1] is a key sensor for IoT applications (e.g., video surveillance), performs onboard analysis of the captured image and video data and only shares the meaningful information with intended devices or end-users. Najjar et al. [11] briefly introduced some basic visual sensor networks (VSN) platforms (Cyclops, MeshEye, Vision Mote, MicrelEye), related architectures and challenges. They further highlighted the need for image processing lightweight algorithms and identified the trade-off between algorithm performance and resource consumption (memory, processing, and power). Winkler and Rinner [12] presented the novel platform TrustEYE.M4 with embedded data security and privacy protection techniques for VSN applications [13]. Birem et al. [14] introduced another FPGA-based smart-camera prototype called DreamCam which was proposed for real-time detection and extraction of visual information.

A stand-alone smart camera performs sensing, processing and communication on a single platform, and various resource-efficient security techniques [6] have been used for data protection. A desirable approach for the protection of the sensed data on a smart camera is the implementation of security techniques close to the sensing unit [15]. The feasibility of data protection at the sensor level is demonstrated with the custom-designed TrustEye.M4 prototype which provides sensor-level privacy protection [16]. The authors further integrated a trusted platform module (TPM) into the camera node and implemented RSA and AES following a sign-then-encrypt approach to ensure security and privacy of data onboard. The limitations of their approach are the significant hardware overhead for resource constrained sensors using TPM and the computational complexity by implementing the security techniques in the sign-then-encrypt way using 2048-bit RSA keys. Another limitation of the TPM-based approach is the invalidation of security proofs if the data modified by an authentic entity for the sake of communication or computation efficiency on the host unit of the smart camera or any later stages.

Haider et al. [17] presented a FPGA-based prototype exploiting physical unclonable functions (PUF) for identity-based signature (IBS) at the sensor level. They further used a certificate-based approach for key generation and implemented the AES-128 and HMAC-SHA256 using the encrypt-then-sign approach to ensure the security of data. The limitations of their approach are the key escrow problem [18] and the overhead of identity-based certification. It is also computationally expensive due to the implementation of encryption and signature in two steps. The AES implementation with a 128-bit key might be vulnerable to attacks. Zhang et al. [19] also proposed a PUF-based CMOS image sensor for sensor-level authentication of the data.

### 2.2. Protection in Smart Camera Networks

The security approaches discussed in the previous section are only applicable to a single smart camera platform (stand-alone node), which entails onboard protection of the sensed data before it leaves the device. In IoT applications usually more than one smart camera is used for the surveillance of a target location. Various efficient techniques [20] for the compression and communication of multimedia data have been proposed for resource constrained environments. For cryptographic approaches [21,22], advanced encryption standard (AES) is considered to be a lightweight security algorithm which is suitable for hardware and software implementation in such environments.

The complexity of security techniques is increasing with increasing size of the smart camera network. A centralized model of the camera network requires large bandwidth and sufficient amount of energy to transfer high volume of data (images/videos) and limits the scalability [23]. An alternative model is a distributed network which reduces the data transfer requirements to a dedicated node, while it does not exploit the advantages of multi-camera networks [24]. A cluster-based model overcomes the limitations of centralized and distributed networks and provides scalability without the risk of single-point failures [25,26]. In a cluster-based network the smart cameras are grouped in distinct clusters, each with a local cluster head. From a security perspective, a cluster head acts like a firewall for the rest of the cluster nodes and reduces the risk of external attacks [27]. In our previous work [9], we proposed aggregate-signcryption for securing cluster-based camera networks while reducing computation and communication overheads.

### 2.3. Use-Cases of Secure Smart Cameras

In video surveillance, a smart camera captures large volumes of data in the form of images or videos and requires efficient security techniques for data protection. Chien et al. [28] used a special node for aggregation of data from video sensors in the IoT-based video surveillance and recommended lightweight algorithms and system-on-chip (SoC) approaches to further improve the computation power of sensors. Alsmirat et al. [29] presented a framework for a secure surveillance system [30]. They used AES for confidentiality and RSA for key distribution. The session key was further secured by HMAC-MD5 hashing and provided authentication and integrity of the video streams. This approach was implemented with the NS-3 simulator and the trade-off between communication delay and security was evaluated. The computation and communication overhead was reduced by encrypting the whole video frame instead of encrypting each data packet. Mora et al. [31] proposed an IoT-based framework for healthcare monitoring and proposed scheduling techniques for sharing the resources among the nodes to reduce the computation costs. This approach preserved local resources for critical processing only. A secure remote authentication of the user was also performed for smart cities applications [32].

A secure and privacy-preserving (SecSPS) IoT framework for smart parking [33] was proposed to find vacant parking places in a city center and monitor the incoming and outgoing vehicles in the parking spots. The SecSPS framework provided the detection and availability of vacant parking locations with real-time guidance to the driver for its reservation. The authors proposed certificate-based RSA key establishment techniques and 128-bit AES encryption with CBC mode for the confidentiality and hashing for data integrity using a sign-then-encrypt approach. Their proposed framework is resilient to various security attacks and ensures data protection and device security for the users. They suggested EC-based certification as alternative for resource limited devices. The SecSPS framework using RSA with 2048 bit and AES with 128 bit in a sign-then-encrypt way is not appropriate for IoT applications due to the large RSA key and security concerns of the weak AES key. Baran et al. [34] used a smart camera for the identification and recognition of vehicles in transportation systems to help law enforcement authorities. Huang et al. [35] presented a security scheme to preserve privacy of parking reservation in an automated valet parking (AVP) application. Their scheme protects the privacy of location and the identity of drivers, and prevents double reservation attacks, where the users can only make a single reservation at the same time. The users can choose the vacant parking place by themselves and the location obfuscation mechanism easily provides location privacy for this use-case. They used a cryptographic approach based on elliptic curve with bilinear pairing and simulated their scheme in Java for comparing the communication and computation overheads with state-of-the-art use-cases.

Won et al. [36] presented a certificateless multi-receiver hybrid encryption scheme for drone-based monitoring services in a one-to-multi-receiver communication scenario, where the drone sends the sensitive data privately to multiple smart objects. The sender re-used a proposed random number to generate the symmetric key used for each receiver. Drones are equipped with a GPU to reduce execution time and optimize the batch verification of signature to speed up the verification procedure. The authors implemented the secure communication protocols on two kinds of medium (equipped with moderate-speed CPU) and high capacity (equipped with CPU as well as GPU) drones for the smart parking and traffic monitoring applications.

Anoop [37] proposes an elliptic curve (EC) cryptosystem with comparatively small key sizes which is more secure than an integer factorization-based RSA cryptosystem. The combination of the public key infrastructure (PKI)-based signing and encryption approach is usually used to guarantee the security properties (e.g., authentication, integrity, freshness and confidentiality) of images or video data in a holistic way. The combination in a sign-then-encrypt way is a two steps process, and its disadvantage is the extra overhead involved in the separate processing of the signature and encryption procedure. Signcryption [7] performs the implementation of signature and encryption in a single step and provides the same level of security with reduced computation and communication overheads.

### 2.4. Signcryption: An Efficient Certificateless Security Approach

In the PKI-based cryptosystem, the certification management for public key authentication is a challenge. Boneh et al. [38] used an identity-based cryptographic approach and eliminated the need for certification, but the limitations of their scheme was the key escrow problem caused by the secret keys generated by a third party such as private key generator (PKG). The key escrow problem was then eliminated by using bilinear pairing-based certificateless cryptography [18]. However, the computation of pairing was not efficient for resource limited devices, so a pairing free approach [39] has been proposed and implemented in a drone-based surveillance application. However, this approach also faces the user revocation problem if a physical attack occurs on the device. In such cases the attackers can access current and future information of the devices.

Pang et al. [40] presented a novel multi-receiver signcryption scheme, and they claimed the complete anonymity preservation of receivers and senders. They also provided public verification and decryption fairness of the data. They multiplied the public key of the sender by a random value to hide the identity of the sender and avoided the cross-comparison and joint conspiracy attacks. Their scheme protects the data from both external and internal attacks. Their scheme is based on the security assumptions of decision bilinear Diffie-Hellman (DHBP) and Gap-BDH approaches. They theoretically evaluated the efficiency of this scheme and proved its security by using a random oracle model.

Niu et al. [41] presented hybrid signcryption which secures multiple messages for multi-receiver in heterogeneous environments. They used different master keys and sent multiple messages from a sender using identity-based cryptography to multiple receivers in a certificateless system. They used hybrid encryption based on a key encapsulation mechanism (KEM) and a data encapsulation mechanism (DEM) to secure the one-time symmetric key along with data. Their approach provides insider security by generalizing the KEM to signcryption KEM and included authentication. They used a PKG and a KGC to calculate the pseudo-identities for the users in their system and generated the partial private keys. They implemented the scheme in the C-programming language by using pairing-based cryptographic library (Libpbc). They proved confidentiality and unforgeability in a random oracle model.

### 2.5. Comparisons with our Approach

Table 1 summarizes the state-of-the-art regarding algorithms, implementation procedures, security properties as well as computational and communication efficiency. In our preliminary work [8], we proposed signcryption and implemented EC-based signature and AES-based encryption in a single step onboard the smart cameras. We then introduced aggregate signcryption [9] to merge signcrypted data within a cluster of smart cameras and to extend the protection to a multi-sender/single-receiver setup. The objective of our proposed work is to generalize protection to multi-sender/multi-receiver setups while maintaining resource efficiency.

We adopted a multi-receiver encryption scheme [46,47] with a sign-then-encrypt approach and customized it to aggregate-signcryption with decryption fairness for multiple receivers. The proposed approach avoids the key escrow problem and does not require a certification for public key authentication. The small key size of EC [48] and the implementation as a signcryption [7] supports real-time data security directly on the smart-camera sensing unit. To the best of our knowledge, our approach is the first deployment of aggregate signcryption for a multi-receiver setup in an IoT context.

## 3. System Architecture

**Figure 1:**
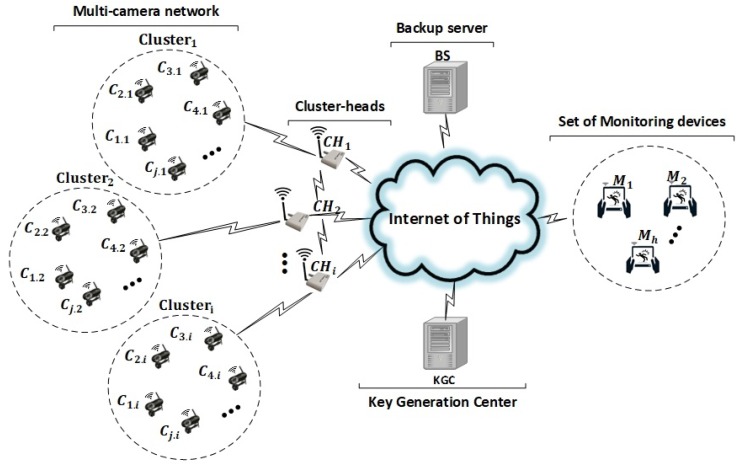
The IoT system architecture. The cluster-based multi-camera network merges individual-signcryptexts into aggregated-signcryptext and provides access to them for multiple monitoring devices. Aggregated-signcryptexts are permanently stored on a backup server. A key generation center provides public parameters and partial private keys for all components of the system architecture.

Figure 1 presents the system architecture and their key components in a typical IoT environment. The key components are smart cameras, cluster heads, monitoring devices, a backup server and a KGC. The smart cameras can detect predefined events due to their local processing capabilities. Once an event has been detected, the camera triggers a description of the event and identifies a region of interest. We group the co-located smart cameras into distinct clusters [25] with a predefined cluster head, which works as a gateway [49] and connects the smart cameras with the rest of the system. The cluster heads and their corresponding smart cameras are labeled as $CH_{i}$ and $C_{j}$ where *i* and *j* represent the identifiers for the cluster head and camera, respectively. Typically, a smart camera has insufficient storage for all captured data due to its resource limitations, so we use a backup server (BS) to permanently store the protected data transferred by the cluster heads for the intended monitoring devices. The backup server provides an authorized access to that stored data for the corresponding monitoring devices ($M_{h}$). A third-party trusted entity KGC is responsible for the partial key generation and public key authentication in our system architecture. The KGC initiates the system setup and key sharing, where the shared keys are used for secure communication in the operational phase of the system.

This architecture generalizes and extends our previous work on securing smart-camera networks for IoT applications [8,9] to a multi-source/multi-receiver scenario, where a group (cluster) of smart cameras provides secure data of detected events to multiple monitoring devices. The key processing steps for such scenario can be summarized as follows: (i) onboard detection of predefined events on the smart cameras within a cluster, (ii) aggregation of the information on the cluster head, (iii) storage of the aggregated information on a backup server and (iv) download of the information which are already stored on the backup server for the respective multiple monitoring devices to complete the surveillance procedure.

**Figure 2:**
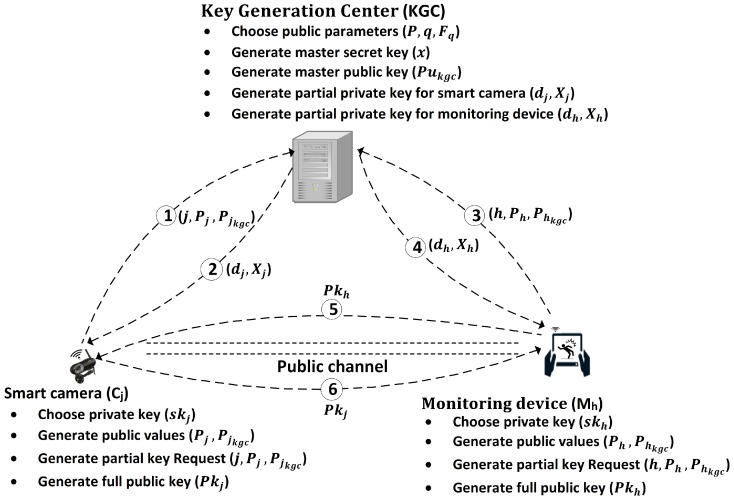
Key generation and distribution in the deployment phase. First, the KGC chooses public parameters and the master secret key and then generates the master public key based on the chosen private key. The smart camera and monitoring device choose their private keys and generate their respective public values. They share their public values with KGC in steps 1 and 3 to request partial private keys. They receive the requested relevant partial private keys in steps 2 and 4 from the KGC, respectively. The smart cameras and monitoring devices generate their full public keys based on their relevant private and partial private keys and share them with each other through a public channel in steps 5 and 6.

Each communicating entity of the system architecture chooses their private key and generates a public value. These entities securely store their private keys and share the public value with the KGC for requesting a partial key. The KGC verifies the request of each entity and then generates a partial key for them. As the requesting entity receives its partial key from the KGC it verifies its authenticity and then generates its full public key. The key generation procedure using the KGC for a smart camera and a monitoring device is shown as in Figure 2.

### 3.1. Requirements and Assumptions

The detail description of the basic requirements for the authentication, integrity, confidentiality, freshness and public verifiability are present in our previous work [8,9], and we extended that for the complex multi-sender/multi-receiver scenarios. We added techniques of exclusive protection and decryption fairness for the decryption and exclusive access of the same data by multiple receivers and further added forward secrecy to maintain the confidentiality of incoming or past data from smart cameras in the case of compromising of a specific session key by an attacker at any stage. We can summarize the requirements for the proposed system architecture as follows: (i) authentication and sharing of the public keys in advance, (ii) exclusive protection of data on smart cameras for different receivers using symmetric keys (exclusive protection on the sender side), (iii) optimization of aggregate signcryption to the multi-receiver scenario, (iv) exclusive access to the received signcrypted data by multi-receiver (exclusive access on the receiver side), (v) maintaining decryption fairness, which is the exclusive decryption of data by monitoring devices using their own session keys, (vi) public verifiability of the data by any trusted or untrusted party and (vii) forward secrecy when session keys are compromised by an attacker.

We assume that each smart camera consists of a trusted sensing unit and a camera host unit [16]. The camera host unit is not explicitly trusted and is responsible for the configuration, management and running of the application and system libraries. The protection of the sensing unit is built upon our previous work [17,45] and has exclusive access to the raw data (images and videos). We assume no explicit protection against denial of service (DoS) attacks on the other components of the system but we can verify the authenticity of incoming requests by using the public parameters and public keys, which is called public verifiability (a property of the signcryption technique). The public verifiability of data can reduce incoming requests of an attacker and only forward authentic requests.

Moreover, we assume that the monitoring device is trustworthy and will generate its private key and keep it secret. Public keys are generated by the smart cameras based on their private and partial private keys and securely share them with the monitoring devices and other components of the system architecture. The KGC generates the partial private keys for all components and can verify and authenticate the public keys of all components within the system architecture.

### 3.2. Threat Model

Smart cameras in IoT applications (e.g., smart home monitoring) have several vulnerabilities [6,50,51], which can be exploited by attackers to gain root access to the smart camera nodes and compromise the security and privacy of data. In such applications the important assets to protect from unauthorized access are the captured sensitive information (images/videos), the secret keys (e.g., private keys and encryption keys) of smart cameras and the camera node itself. The open infrastructure (e.g., Internet) in IoT applications pose a challenge to mitigate such attacks and to secure the smart cameras from unauthorized access. In the proposed system architecture (Figure 1) an attacker may get access to the camera host part, cluster head, communication channel and backup server. The attacker can compromise the integrity and alter the data while remain undetected. Another capability of the attackers is to compromise the authenticity of source and insert their own information by using the identity of smart cameras. In a multi-receiver scenario any trusted monitoring device can verify the protected data which are intended for another monitoring devices.

We consider all these security threats and present a cluster-based secure approach for the system architecture to reduce the risks of attacks in open infrastructure. We present certificateless multi-receiver aggregate-signcryption techniques with public verifiability, decryption fairness, and forward secrecy to avoid the key escrow problem and guarantee the protection, verification, and exclusive access to the data only by authentic/intended users. The details of the proposed solution are presented in the following section.

## 4. Proposed Solution

In this section, we present lightweight security techniques to protect and secure the sensitive information in the proposed system architecture. The design goals of the security techniques are mainly derived from the considered case studies and can be summarized as: (i) to allow only the authentic requests, (ii) to reduce the transmission of unnecessary data, (iii) to protect the captured information from unauthorized access throughout its lifetime, and (iv) to prove the authentication and integrity of the information on the intended monitoring devices. The scheme provides data security and decryption fairness to multiple monitoring devices in the proposed system architecture. The proposed techniques can also be used to provide data security for related smart camera applications such as intelligent surveillance (e.g., [52,53]) or safety monitoring (e.g., the automatic detection of cracks in buildings, bridges and subways tunnels [54]).

### 4.1. Overview of Security Techniques

We implement our proposed security techniques in two phases. The deployment phase performs the key generation and key sharing with authentication for smart cameras and monitoring devices. In this phase the KGC defines the system setup, chooses the public parameters, and generates the partial keys for all participating devices (Figure 2). The full private and public keys are defined by the smart cameras and monitoring devices themselves to avoid the key escrow problem.

**Figure 3:**
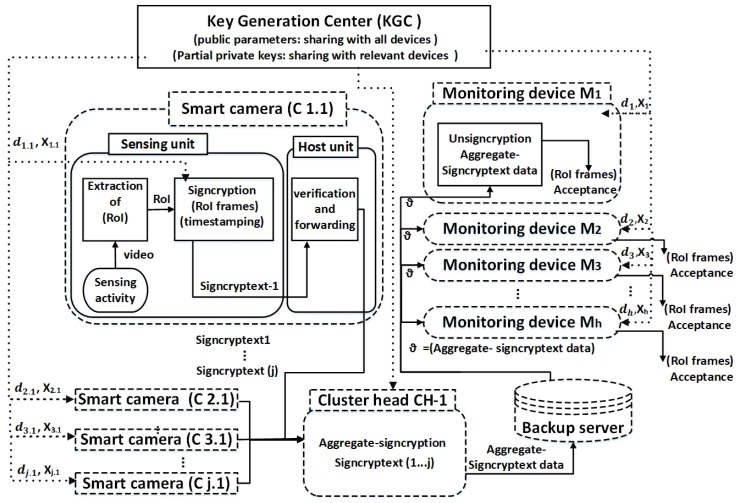
Operational phase depicting the processing flow of aggregate-signcryption for smart cameras in cluster *i* sending protected data to multiple monitoring devices (multi-receiver). The left side shows multi-cameras (sender devices) and right side shows the monitoring devices (multi-receiver). The distribution of keys and public parameters is shown by dotted lines, and the transfer of actual data is shown by solid lines, where *d* and *X* represent the partial keys and ϑ represents the aggregate-signcryptext data for multi-receiver (monitoring devices).

In the operational phase, the smart camera uses their private key, the public keys of receiving devices and the public parameters which are already defined by the KGC to execute the signcryption (Figure 3). We adopt the multi-receiver encryption approach [46,47] to perform the signcryption procedure. We then apply aggregation on the cluster head to merge the signcrypted data into a single, compact packet. The aggregate-signcryptext data is then sent to the backup server, from where the monitoring device can access and download it. The monitoring devices first check the authenticity and verification of data and then proceed with the decryption by using their relevant decryption keys. The decryption keys are extracted from the received aggregate-signcryptext packet by each monitoring device exclusively.

### 4.2. Deployment Phase

In the deployment phase, the KGC defines the system architecture and security parameters (e.g., EC type, keys length and parameters) in advance, which reduce the load on the resource constrained devices during the operational phase. The KGC is also responsible for the generation of partial private keys for all participating devices. In the deployment phase, the smart cameras are grouped into distinct clusters, and the numbers of relevant monitoring devices for each cluster are defined. The KGC initializes the system setup and generates the partial private keys for all participating devices on request, where each device further defines their full public key which is partially depending on the partial private key which is already received from the KGC. The processing steps of key generation and distribution are shown in Figure 2 and explained in the following section.

#### 4.2.1. Preliminary Setup for Generation of Security Parameters by KGC

A KGC generates and shares the public parameters of the system, which are used by the participating devices to define their own security setup. The EC base point *P*, finite field $F_{q}$, prime number *q*, and the required characteristics (e.g., the type and length of keys) are included as public parameters in the preliminary setup [37]. These security parameters are fixed and generated in advance by the KGC during the deployment phase. The KGC shares these security parameters with all participating devices of the system for further use in the operational phase. A KGC defines the preliminary setup as follows:

The KGC takes $k\in Z_{q}^{+}$ as input and generates the public parameters and chooses its master secret key. An EC over the finite field $F_{q}$ is represented by $E(F_{q})$ with a base point $P\in F_{q}$. The parameter *q* is a prime number specifying the finite field $F_{q}$. The KGC generates its master secret key and the public parameters for the system as follows:

- Use the preliminary setup and determine the public parameters
- Choose the master key $x\in Z_{q}^{*}$ uniformly at random and compute the system public key $Pu_{{}_{kgc}}=xP$
- Choose the cryptographic hash functions $H_{1}:{\left\{0,1\right\}}^{*}\times G_{1}\times G_{1}\to Z_{q},H_{2}:G_{1}\to {\left\{0,1\right\}}^{k_{0}},H_{3}:{\left\{0,1\right\}}^{*}\to Z_{q}$ and $H_{4}:{\left\{0,1\right\}}^{k_{0}}\to {\left\{0,1\right\}}^{k}$, where *k* shows the fixed key length of a symmetric key and $G_{1}$ is a cyclic group generated by using the EC base point *P*.
- Keep the master key *x* as secret and publish the public parameters along with $Pu_{{}_{kgc}}$.

In the proposed system architecture, the smart cameras (senders) and the monitoring devices (receivers) share their public parameters with each other. We only present the key generation algorithms of smart cameras for the sake of simplicity. The same key generation procedure applies for the monitoring devices. We also provide a description of the used symbols in Table A1.

#### 4.2.2. Request of Partial Private Key by a Smart Camera

Each smart camera *j* in the system architecture randomly chooses a secret value $sk_{j}\in Z_{q}^{*}$ and generates two public values using the base point *P* and the master public key $Pu_{{}_{kgc}}$ of KGC, e.g., $P_{j}=x_{j}.P$ and $P_{j_{kgc}}=x_{j}.Pu_{{}_{kgc}}$. Then the smart camera sends the identity and public values ($j,P_{j},P_{j_{kgc}}$) to the KGC to request a partial private key.

#### 4.2.3. Partial Private Key Generation by KGC

Once the KGC receives the request from the smart camera for partial key generation, it first verifies its validity by checking if $P_{j_{kgc}}=xP_{j}$. If the validity is true then the KGC generates a partial private key, otherwise it rejects the request. The KGC generates the partial key by using its master secret key *x*, the identity *j* of the smart camera and the public parameters with permitted time period $t_{j}$ as follows:

- Choose $r_{j}\in Z_{q}^{*}$ randomly for each smart camera,
- Compute $X_{j}=r_{j}.P$, $P_{x}=P_{j}+X_{j}$ and
- Compute $d_{j}=H_{1}\left(j,P_{j},P_{x}\right)x+r_{j}$mod*q*.

The KGC sends the partial private key $\left(d_{j},X_{j}\right)$ to smart camera *j* via a secure channel.

#### 4.2.4. Public Key of Smart Camera

As the smart camera receives its partial private key $\left(d_{j},X_{j}\right)$ from the KGC, it first verifies its validity and then generates the full public key as follows:

- Compute $P_{j}^{\prime }=P_{j}+X_{j}$
- Compute $H_{j}=H_{1}\left(j,P_{j},P_{j}^{\prime }\right)$ and check if $d_{j}P=H_{j}Pu_{{}_{kgc}}+X_{j}$ according to the Schnorr digital signature [55,56]. Otherwise, reject the partial private key
- Compute $P_{j}^{\prime \prime }=H_{j}^{-1}P_{j}^{\prime }$

The smart camera *j* chooses the full public key as $Pk_{j}=\left(j,P_{j},P_{j}^{\prime },P_{j}^{\prime \prime }\right)$.

### 4.3. Operational Phase

In the operational phase each smart camera initiates the signcryption process for the intended monitoring devices. The smart camera uses its private key $sk_{j}$ for the signature part, generates a session key for the encryption part and performs signcryption on the captured data as described in the following subsections.

#### 4.3.1. Session Keys Generation

Each smart camera of a cluster participating in the surveillance of a specific area generates the symmetric keys for the encryption of data intended for the distinct monitoring devices as follows.

- The smart camera *j* chooses the public parameters and the list *l* of the identities of the monitoring devices.
- The smart camera *j* generates the symmetric key $K_{enc}$ and the internal state information (e.g., firmware version and timestamp of the health status) represented by ω, using the public keys and other identity information of the monitoring devices.

The identity of smart camera *j*, the full public key $pk_{j}$, the full private key $sk_{j}$, the monitoring device identity *h*, the permitted time period $t_{h}$ and the full public key $pk_{h}$ are given as inputs to the session key generation. The smart camera performs the following steps to get the symmetric key $K_{enc}$ for all monitoring devices of a list *l*:

- The smart camera chooses $s_{j}\in Z_{q}^{*}$ and $\sigma \in {\left\{0,1\right\}}^{k}$ randomly
- The smart camera then generates the session key as $K_{enc}=H_{4}\left(\sigma \right)$

#### 4.3.2. Multi-Receiver Signcryption by Smart Cameras

Each smart camera signcrypts the region of interest $\left({RoI}_{frames(f)}\right)$ of frames *f* for the list *l* of monitoring devices with the relevant encryption key $K_{enc}$ as follows:

- The region of interest is encrypted as $\varrho \left(l\right)=Enc_{K_{enc(l)}}\left({RoI}_{frames(f)}\right)$.
- The output of the required ciphertext for list *l* of monitoring devices is given as θ=(ϱ(1),ϱ(2),ϱ(3),⋯ϱ(l)).

Each smart camera uses the ciphertext data θ to complete the signcryption procedure with the following steps:

(1) $$k_{l}=v.P$$

(2) $$r=H_{3}(\theta ∥t_{j}∥k_{l}∥\omega_{l}∥l)$$

(3) $$a_{j}=(H_{j}^{-1}d_{j}+rsk_{j}+s_{j})\;mod\;q$$

(4) R=r.P

(5) $$H_{h}=H_{1}\left(h,P_{h},P_{h}^{\prime }\right)$$

(6) $$U_{h}=rH_{h}\left(Pu_{{}_{kgc}}+P_{h}^{\prime \prime }\right)$$

(7) $$V=\sigma ⊕H_{2}\left(R\right)$$

(8) $$Signcryptext_{j}=(a_{j},U_{1},U_{2}\ldots ,U_{h},V,\theta ,l,k_{l},\omega_{l},t_{j})$$

#### 4.3.3. Multi-Receiver Aggregate-Signcryption

For multi-receiver aggregate-signcryption, the cluster head $CH_{i}$ performs the aggregation of all signcryptexts [9] from the smart cameras with other parameters for the sorting of relevant data. The sorting of $signcryptext_{j}$ is perform according to the identities *h* with relevant public keys $pk_{h}$ of the list *l* of monitoring devices. The cluster head verifies each signcryptexts with the public verification method as described in Section 4.3.4 and then uses the public keys of the smart cameras and monitoring devices to generate the aggregate-signcryptext as follows:

- Compute $S=\sum_{j=1}^{n}a_{j}$ and parse the θ according to the $l,K_{l},\omega_{l},t_{j}$ in a specific order.
- Merge the signcrypted data $\left(\theta \left(1\right)\cdots \theta \left(l\right),U_{1}\cdots U_{h},l,k_{l},\omega_{l},t_{j},S\right)$ to the aggregate-signcryptext (ϑ) form.

#### 4.3.4. Unsigncryption by Monitoring Devices

Unsigncryption is performed on the monitoring devices of the aggregate-signcryptexts after sorting them according to the list *l*. If the authentication and integrity of the data is verified, the decryption procedure is then applied to the given ciphertexts θ=(ϱ(1),ϱ(2),ϱ(3),⋯ϱ(l)) on each monitoring device. The decryption algorithm requires the full private and public keys of the monitoring devices with the public keys of the smart cameras to retrieve the decryption keys of intended monitoring devices. The monitoring devices use the public keys of the smart camera $Pk_{j}=\left(j,P_{j},P_{j}^{\prime },P_{j}^{\prime \prime }\right)$ for the acceptance and verification of the signcrypted data.

- Find the corresponding $U_{h}$ from the list *l* of the $signcryptext_{j}$.
- Compute $r^{\prime }=H_{3}(\theta ∥t_{j}∥k_{l}∥\omega_{l}∥l)$.
- Compute $k_{l}^{\prime }=a_{j}P-\left(\left(r^{\prime }-H_{j}^{-1}\right)P_{j}+P_{j}^{\prime \prime }+Pu_{{}_{kgc}}\right)$.
- Accept the signcrypted data, if $k_{l}^{\prime }=k_{l}$ and then verify the integrity of data ($U_{h}=r^{\prime }H_{h}\left(Pu_{{}_{kgc}}+P_{h}^{\prime \prime }\right)$).
- Proceed with the decryption if the data acceptance and verification was successful.
- Compute $R^{\prime }={\left(d_{h}+sk_{h}\right)}^{-1}U_{h}$.
- Compute $\sigma^{\prime }=V⊕H_{2}\left(R^{\prime }\right)$.
- Compute decryption key $K_{dec(l)}=H_{4}\left(\sigma^{\prime }\right)$.
- Decrypt the ciphertext data to plaintext $RoI_{{frames}_{f}}=Dec_{k_{dec(l)}}$ (θ) or apply decryption to the parsed ciphertexts e.g., $Dec_{k_{dec(l)}}$(θ(l))$=Dec_{k_{dec(l)}}\left(\varrho \left(1\right),\varrho \left(2\right),\varrho \left(3\right),\cdots \varrho \left(l\right)\right)$ data.

#### 4.3.5. Correctness of the Scheme

As described in the unsigncryption process in Section 4.3.4, the $R^{\prime }$ value is needed to recover the relevant decryption keys on the monitoring devices for the decryption of the authentic ciphertexts. Only those monitoring devices can recover the decryption keys whose public information were already used in the aggregate-signcryption process, i.e., for the computation *R* in Equation (4). Therefore, now only those monitoring devices can use their associated private keys. The recovery of the correct decryption keys is based on the authentication and integrity of the aggregate-signcryptexts and on the associated private keys of the monitoring devices. These $R^{\prime }$ and *R* values should be equal to recover the correct decryption keys for the decryption of ciphertexts θ. The relevant monitoring devices prove the data integrity and authentication during unsigncryption by computing $k_{l}^{\prime }$ and then $r^{\prime }$. The $r^{\prime }$ value is further used in the computation of $U_{h}$ which provides $R^{\prime }$ (e.g., $R^{\prime }={\left(d_{h}+sk_{h}\right)}^{-1}U_{h}$). Therefore, the proof of $R^{\prime }=R$ can be described as follows:

$$\begin{array}{cc} R^{\prime } & ={\left(d_{h}+sk_{h}\right)}^{-1}U_{h}\\ & =\frac{U_{h}}{\left(d_{h}+sk_{h}\right)}\\ & =\frac{rH_{h}\left(Pu_{{}_{kgc}}+P_{h}^{\prime \prime }\right)}{\left(H_{1}\left(h,P_{h},P_{h}^{\prime }\right)x+x_{h}\right)+sk_{h}}\\ & =\frac{r(H_{h}\left(Pu_{{}_{kgc}}\right)+H_{h}.H_{h}^{-1}P_{h}^{\prime })}{\left(H_{1}\left(h,P_{h},P_{h}^{\prime }\right)x+x_{h}\right)+sk_{h}}\\ & =\frac{r(H_{h}\left(Pu_{{}_{kgc}}\right)+\left(PK_{h}+P_{h}\right))}{\left(H_{1}\left(h,P_{h},P_{h}^{\prime }\right)x+x_{h}\right)+sk_{h}}\\ & =\frac{r(H_{1}\left(h,P_{h},P_{h}^{\prime }\right)xP+\left(sk_{h}+x_{h}\right)P)}{H_{1}\left(h,P_{h},P_{h}^{\prime }\right)x+x_{h}+sk_{h}}\\ & =\frac{r(H_{1}\left(h,P_{h},P_{h}^{\prime }\right)x+sk_{h}+x_{h})P}{H_{1}\left(h,P_{h},P_{h}^{\prime }\right)x+sk_{h}+x_{h}}\\ & =rP \end{array}$$

The $R^{\prime }$ value is further used in the computation of $\sigma^{\prime }$ that should be equal to the value of σ (which was already computed for the generation of encryption keys on smart cameras side). The correctness can thus be shown as follows:

$$\begin{array}{cc} \sigma^{\prime } & =V⊕H_{2}\left(R^{\prime }\right)\\ & =\sigma ⊕H_{2}\left(R\right)⊕H_{2}\left(R^{\prime }\right)\\ & =\sigma ⊕H_{2}\left(rP\right)⊕H_{2}\left(rP\right)\\ & =\sigma \end{array}$$

Hence, the list of decryption keys can be recovered on each monitoring device according to their own information as follows:

$$\begin{array}{cc} K_{dec(l)} & =H_{4}\left(\sigma^{\prime }\right)\\ & =H_{4}\left(\sigma \right)\\ & =K_{enc(l)} \end{array}$$

Data authentication and replay attack prevention can be checked as follows: First, calculate the value of $a_{j}P$.

$$\begin{array}{cc} hj & =H_{1}\left(j,P_{j},P_{j}^{\prime }\right)\\ a_{j}P & =H_{j}^{-1}d_{j}P+Pu_{{}_{kgc}}P+vP\\ & =H_{j}^{-1}\left(H_{j}sP+x_{j}P\right)+rP_{j}+vP\\ & =xP+H_{j}^{-1}x_{j}P+rP_{j}+vP\\ & =Pu_{{}_{kgc}}+H_{j}^{-1}x_{j}P+rP_{j}+vP \end{array}$$

Second, calculate the value of $\left(r^{\prime }-H_{j}^{-1}\right)P_{j}+P_{j}^{\prime \prime }+Pu_{{}_{kgc}}$:

$$\begin{array}{cc} \left(r^{\prime }-H_{j}^{-1}\right)P_{j}+P_{j}^{\prime \prime }+Pu_{{}_{kgc}} & =\left(r^{\prime }-H_{j}^{-1}\right)P_{j}+H_{j}^{-1}\left(P_{j}+x_{j}P\right)+Pu_{{}_{kgc}}\\ & =r^{\prime }P_{j}+H_{j}^{-1}x_{j}P+Pu_{{}_{kgc}} \end{array}$$

Finally, the value of $\left(r^{\prime }-H_{j}^{-1}\right)P_{j}+P_{j}^{\prime \prime }+Pu_{{}_{kgc}}$ must be subtracted from $a_{j}P$ resulting in $k_{l}$, which shows the authenticity and proof of the prevention of the replay attack:

$$\begin{array}{cc} a_{j}P-\left[\left(r^{\prime }-H_{j}^{-1}\right)P_{j}+P_{j}^{\prime \prime }+Pu_{{}_{kgc}}\right] & =vP\\ & =vP\\ & =k_{l} \end{array}$$

### 4.4. Security Analysis

The multi-receiver aggregate-signcryption scheme for the proposed system architecture (Figure 1) provides the basic security properties, e.g., public verifiability, authentication, integrity, freshness, confidentiality, decryption fairness and forward secrecy for the captured data received from smart cameras. These properties are briefly analyzed in the following sections.

#### 4.4.1. Public Verification

The security technique of multi-receiver aggregate-signcryption provides the public verifiability of the data by any trusted or untrusted entity in the system without decryption of the data. The public verifiability proves, if $U_{j}=r^{\prime }H_{j}\left(Pu_{{}_{kgc}}+P_{j}^{\prime \prime }\right)$ is true for the smart camera *j*. The advantage of the public verifiability is that the authenticity of the data received from the source can be proven at any stage by a trusted or untrusted entity. The public verification does not require the private keys of smart cameras, the verification is possible with the relevant public information of the devices.

#### 4.4.2. Authentication and Integrity

The authentication can be checked by the intended receiver by computing $k_{l}^{\prime }=a_{j}P-\left(\left(r^{\prime }-H_{j}^{-1}\right)P_{j}+P_{j}^{\prime \prime }+Pu_{{}_{kgc}}\right)$ and then comparing it with the received value of $k_{l}$ from the smart camera. $k_{l}^{\prime }=k_{l}$ means that the data comes from an authentic smart camera. The integrity of the received data can also be verified by $k_{l}^{\prime }=k_{1}$ because in the computation of $k_{l}$ the value of $r=H_{3}(\theta ∥t∥k_{l}∥\omega_{l}∥l)$ is required, which is the hashed valued of the ciphertext data θ along with the other information. The *r* value is further multiplied with the secret key of smart camera ($sk_{j}$), as its public key is used for the verification purpose in the computation of $k_{l}^{\prime }$. Therefore, the proof for $k_{l}^{\prime }=k_{l}$ provides both the properties of integrity and authenticity of the received data. The attacker cannot compromise the integrity and authenticity without the private key of smart camera $sk_{j}$ and guessing of a private key from public key is hard problem because of the elliptic-curve-based discrete logarithm problem (ECDLP) assumption.

#### 4.4.3. Decryption Fairness and Confidentiality

The decryption fairness is the capability of the monitoring device to extract the decryption key from shared information using their own credentials. Confidentiality of data is the prevention of access from unauthorized users and the guarantee of exclusive access for the intended receivers. Only the intended receivers can exclusively access the shared information for their own decryption key recovery with the help of their private keys. None of the monitoring devices other than intended can recover the decryption key to access the data for another monitoring device, because the private key associated with the public keys is hard to guess due to the assumption of ECDLP.

#### 4.4.4. Freshness

The smart camera uses the hash of the timestamp in the computation of *r* along with the concatenated value of the ciphertext. This guarantees the freshness of the ciphertext data. If the timestamp value is compromised by an attacker, then the computation of $r^{\prime }$ on monitoring device results in an incorrect value, and the authenticity and integrity proof fails.

## 5. Experimental Evaluation

In this section, we present the experimental setup and investigate the computational effort. In the deployment phase, we measure the computation time of key generation and verification. In the operational phase, we measure and compare the computation times and communication overheads for the individual-signcryption, aggregate-signcryption and multi-receiver aggregate-signcryption approaches.

### 5.1. Experimental Setup

We have prototypically implemented our system architecture (Figure 1), where standard laptops (Intel core i5 with 2.6 GHz and 8 GB RAM) running Windows 10 serve as platform for the key devices (e.g., smart cameras, cluster head, monitoring devices and KGC). We used a standard laptop for ease of implementation and fair comparisons of the different approaches. Runtime measurements for individual- and aggregate-signcryption have been performed on embedded platforms in our previous work [8,9].

In our experiments each camera signcrypts 25 images, where each image has a predefined QVGA resolution (320×240 pixels) and a size of 30 kB. The open source library BouncyCastle [57] is used for the implementation of the EC-based signcryption algorithm and used the EC-finite field of P-384 and a 256 bit AES key. The proposed techniques are implemented in Java due to readily available libraries for the main cryptographic building blocks. We run the application with the same configuration ten times for key generation, signcryption, aggregate-signcryption and multi-receiver aggregate-signcryption for each device and recorded their average running time.

### 5.2. Deployment Phase

In the deployment phase, the KGC initiates the system setup and shares the public parameters among all participating devices (i.e., the smart cameras and monitoring devices). Each device uses those public parameters, chooses a private key, and sends a request for partial key generation to the KGC. The KGC verifies the request and generates a partial key for the requesting device. After the KGC has sent the partial key to the requesting device, it first verifies its authenticity. The requesting device further generates a full public key based on their partial private key and public parameters. The computation times of these steps are summarized in Table 2. The KGC only requires 20.2 ms for its public key generation while the smart cameras and monitoring devices require more time because of their full public key generation based on their partial and private keys. The total computation time required to generate a full public key with the help of the KGC using a certificateless approach is 217.8 ms on a smart camera, 217.2 ms on a monitoring device and 99.5 ms on the KGC platform.

### 5.3. Operational Phase

In the operational phase, the smart cameras monitor a specific area, capture relevant information in the form of images and perform signcryption to secure it for a single device or multiple monitoring devices. We evaluate the computation time and communication overheads for individual-, aggregate- and multi-receiver aggregate-signcryption with different numbers of senders (*m* smart cameras) and receivers (*n* monitoring devices). We measure the computation times of the individual steps of each approach and compare the total runtimes based on three scenarios: single-sender/single-receiver (1-1), multiple-sender/single-receiver (*m*-1) and multiple-senders/multiple-receivers (*m*-*n*).

#### 5.3.1. Computation Time of Individual-Signcryption

Table 3 depicts the measured computational times for the key steps of individual-signcryption: signcryption, verification and decryption. These times have been measured for five different cases (C1 to C5). Here each camera individually signcrypts the captured images and transfers them to the monitoring device where each signcrypted data is verified and decrypted sequentially. In the case of multiple senders, the cameras operate in parallel, thus the maximum signcryption time limits the total time on the sender. On the receiver, the received signcrypted data must be processed sequentially. In the case of multiple receivers, each camera must separately signcrypt the images for each receiver. The total time for individual-signcryption can be estimated as follows

(9) $$TT=n\cdot \left(max_{m}\left(ST\right)\right)+max_{n}\left(m\cdot \left(VT+DT\right)\right)$$

where $max_{m}$ and $max_{n}$ represent the longest time among *m* smart cameras and *n* monitoring devices, respectively.

#### 5.3.2. Computational Time of Aggregate-Signcryption

Table 4 depicts the measured computational times for the key steps of aggregate-signcryption: signcryption, aggregation, verification and decryption. These times have been measured in a cluster of five cameras that send their signcrypted images to the cluster head for aggregation. The cluster head then transfers the aggregated data to the monitoring device where only a single verification is necessary. In the case of multiple receivers, still each camera separately signcrypts the images for each receiver. Thus, the total time for aggregate signcryption can be estimated as follows

(10) $$TT=n\cdot \left(max_{m}\left(ST\right)+AT\right)+max_{n}\left(VT+m\cdot DT\right).$$

#### 5.3.3. Computational Time of Multi-Receiver Aggregate-Signcryption

Table 5 depicts the measured computational times for the key steps of multi-receiver aggregate-signcryption: signcryption, aggregation, verification and decryption. These times have also been measured in a cluster of five cameras. Please note that in this approach, signcryption and aggregation is more complex than in the other approaches but no separate signcryption is required for each receiver in the case of multiple monitoring devices. Thus, the total time can be estimated as follows

(11) $$TT=max_{m}\left(ST\right)+AT+max_{n}\left(VT+m\cdot DT\right).$$

#### 5.3.4. Performance Comparison

Table 6 compares the total times of our three approaches based on three scenarios: one sender and one receiver (1-1), five senders and one receiver (5-1) and five senders and three receivers (5-3). The total times are based on the measured run times of the corresponding approaches and Equations (9)–(11). We highlighted the most efficient approach for each scenario in gray.

As expected, individual-signcryption is most efficient for the 1-1 scenario due to the low overhead. Aggregate-signcryption is superior for the 5-1 scenario, since it avoids multiple verifications on the receiver. Finally, multi-receiver aggregate-signcryption is the best option for scenario 5-3, where it shows a reduction of 32.89% and 28.90% as compared to individual-signcryption and aggregate-signcryption, respectively.

### 5.4. Communication Efficiency

Table 7 presents the communication efficiency of our three approaches by comparing the total amount of transferred data and the number of necessary data transfers. In our experimental setup, each smart camera signcrypts 25 images which a total size of 750 kB. We use the AES 256 bit encryption scheme in CBC mode which results in a ciphertext of same size as the input data.

In the 1-1 scenario, the individual-, aggregate- and multi-receiver aggregate-signcryption transfer 750 kB of ciphertext data. The individual- and aggregate-signcryption require extra data of 72 Bytes for the signature part, while multi-receiver aggregate-signcryption requires extra data of 340 Bytes, because additional parameters are needed for the verification of the signature to enable the multi-receiver setup. Aggregate-signcryption and multi-receiver aggregate-signcryption require an additional transfer to the cluster head.

In the 5-1 scenario, the five smart cameras send their protected data to a single monitoring device, so the individual-, aggregate-, and multi-receiver aggregate-signcryption send the same amount of ciphertext data (e.g., 3750 kB), while the size of the extra data varies for each case. Individual-signcryption requires 360 Bytes for the individual verification of the signcryptexts. Aggregate-signcryption performs only a single verification and reduces the extra data to 168 Bytes. Multi-receiver aggregate-signcryption requires 340 Byte to enable the decryption of same data for multiple monitoring devices (which are actually not required in a single-receiver scenarios). Aggregate- and multi-receiver aggregate-signcryption require five transfers to the cluster head and one transfer to the monitoring device or backup server.

In the 5-3 scenario, the individual- and aggregate-signcryption protect the same data three times for the three different monitoring devices which aggregates to 11,250 kB of ciphertext data. Similarly, extra data must be separately included for each receiver. However, multi-receiver aggregate-signcryption sends the same ciphertext to all monitoring devices and needs only 24 Bytes for each monitoring device in addition to the single-receiver extra data. Similar to the computational efficiency, individual-signcryption is most communication efficient for the 1-1 scenario due to the low overhead. Aggregate-signcryption is superior for the 5-1 scenario, since it avoids multiple signatures and verifications. Finally, multi-receiver aggregate-signcryption is the best option for scenario 5-3.

## 6. Conclusions

In this paper, we investigated certificateless key generation technique and lightweight multi-receiver aggregate signcryption for cluster-based smart camera IoT applications. First, we adopted EC-based signcryption for each smart camera to achieve end-to-end lifetime data security. Second, we implemented aggregation on cluster heads to merge the signcryptexts as a multi-receiver aggregate-signcryptext packet and to avoid the transfer of unnecessary extra data. Third, we performed unsigncryption on each monitoring device with public verifiability and exclusive access to the encrypted data. Finally, in our experimental evaluation we explored the computation and communication effort of individual-, aggregate- and multi-receiver aggregate-signcryption on three different sender/receiver scenarios.

We foresee several directions for future work including investigating alternative encryption approaches for signcryption, implementation on embedded smart camera platforms and deployment in smart home case study.

## Figures and Tables

**Table 1:** Comparison with state-of-the-art approaches. Legend: CL: certificateless, A: authenticity, I: integrity, C: confidentiality, DF: decryption fairness, PV: public verifiability, Au: authorization, CP: computation, CM: communication, Sc: scalability, PA: proposed approach.

Ref.	Algorithms	Implementation Procedure		Security Properties						M-to-M Efficiency		
		Approach	CL	A	I	C	DF	PV	Au	CP	CM	Sc
[36]	eCLSC, CLDA	agg.signc, TKEM/DEM	✓	✓	✓	✓	1	✓	1	✗	✗	M-1
[42]	KCDSA, SKE	signcryption	✓	✓	✓	✓	1	✓	1	✗	✗	1-1
[43]	ECDSA, SKE	IBC, DH, signcryption	✗	✓	✓	✓	1	✗	1	✗	✗	1-1
[44]	AES, BLS	abf-agg.signcryption	✗	✓	✓	✓	1	✓	1	✗	✗	M-1
[45]	RSA, AES	sign-then-encrypt	✗	✓	✓	✓	1	✗	1	✗	✗	1-1
[17]	HMAC, AES	encrypt-then-sign	✗	✓	✓	✓	1	✗	1	✗	✗	1-1
[33]	RSA, AES	sign-then-encryp	✗	✓	✓	✓	1	✗	1	✗	✗	1-1
[8]	ECDSA, AES	signcryption	✗	✓	✓	✓	1	✓	1	✗	✗	1-1
[9]	ECDSA, AES	agg-signcryption	✗	✓	✓	✓	1	✓	1	✗	✗	M-1
PA	EC-ShDSA, AES	agg-signcryption	✓	✓	✓	✓	>1	✓	>1	✓	✓	M-M

**Table 2:** Keys generation and verification time (in ms) in the deployment phase. Legend: SC: smart camera, MD: monitoring device, KGC: key generation center, Pa: partial, Pu: public, TT: total time. The symbol—indicates that the required action is not performed on the corresponding device for the key generation or verification.

	Computational Time (all in [ms])					
Devices	Generation Algorithm			Verification Algorithm		TT
	Pa-Key-Request	Pu-key	Pa-Key	Pa-Key	Pa-Key-Request	
SC	100.7	85.2	–	31.9	–	217.8
MD	100.3	84.7	–	32.2	–	217.2
KGC	–	20.2	47	–	32.3	99.5

**Table 3:** Computational time for individual-signcryption. Legend: SC: smart camera, MD: monitoring device, ST: signcryption time, VT: verification time, DT: decryption time.

Id	SC	MD	
	ST [ms]	VT [ms]	DT [ms]
C1	320.5	155.4	283.8
C2	321.0	154.7	285.0
C3	319.8	156.0	284.7
C4	321.3	155.3	286.2
C5	320.7	154.8	283.9

**Table 4:** Computational time for aggregate-signcryption. Legend: SC: smart camera, CH: cluster head, MD: monitoring device, ST: signcryption time, AT: aggregation time, VT: verification time, DT: decryption time.

Id	SC	CH	MD	
	ST [ms]	AT [ms]	VT [ms]	DT [ms]
C1	320.7	145.5	160.3	284.9
C2	321.2			285.0
C3	319.9			286.3
C4	321.1			284.9
C5	322.0			285.4

**Table 5:** Computational time for multi-receiver aggregate-signcryption. Legend: SC: smart camera, CH: cluster head, MD: monitoring device, ST: signcryption time, AT: aggregation time, VT: verification time, DT: decryption time.

Id	SC	CH	MD	
	ST [ms]	AT [ms]	VT [ms]	DT [ms]
C1	345.4	166.2	172.4	288.3
C2	346.0			287.7
C3	345.2			286.9
C4	344.9			288.5
C5	345.5			287.6

**Table 6:** Comparisons of total times (in ms) of different approaches for one smart camera/one monitoring device (1-1), five smart cameras/one monitoring device (5-1) and five smart cameras and three monitoring devices (5-3).

Scenario	Individual-Signcryption	Aggregate-Signcryption	Multi-Receiver Aggregate-Signcryption
1-1	759.7	911.4	972.3
5-1	2521.1	2054.3	2123.6
5-3	3166.6	2989.0	2124.9

**Table 7:** Comparisons of communication efficiency in terms of transferred data and number of data transfers of different approaches for one smart camera/one monitoring device (1-1), five smart cameras/one monitoring device (5-1) and five smart cameras/three monitoring devices (5-3). Legend: SD: signcryptext data, CD: ciphertext data, ED: extra data for signature and verification, NT: number of transfers.

Scenario	Individual-Signcryption			Aggregate-Signcryption			Multi-Receiver Aggregate-Signcryption		
	SD		NT	SD		NT	SD		NT
	CD [kB]	ED [B]		CD [kB]	ED [B]		CD [kB]	ED [B]	
1-1	750	72	1	750	72	2	750	340	2
5-1	3750	360	5	3750	168	6	3750	340	6
5-3	11,250	1080	15	11,250	504	18	3750	388	8

## Appendix A

**Table A1:** Notation.

*Symbol*	*Description*
$k,k_{0}$	Bit length of security parameters
*q*	Large prime number
*P*	Base point of EC of order *q*
$F_{q}$	Finite field over prime number *q*
$E(F_{q})$	Elliptic curve over $F_{q}$
$Z_{q}^{+}$	Set of additive integers over mod *q*
$Z_{q}^{*}$	Set of multiplicative integers over mod *q*
$x\in Z_{q}^{*}$	Master secret key chosen by KGC
$Pu_{{}_{kgc}}$	Master public key of KGC
$G_{1}$	Group field generated by EC with base point *P*
$H_{1},H_{2},H_{3},H_{4}$	One-way and collision resistant hash functions
${\left\{0,1\right\}}^{*}$, ${\left\{0,1\right\}}^{k}$	Input space of arbitrary and fixed length, respectively
$x_{j}$, $x_{h}$	Secret value of smart camera and monitoring device, respectively
$r_{j}\in Z_{q}^{*}$	Secret value chosen by KGC for partial key generation of smart camera *j*
$\left(d_{j},X_{j}\right)$	The concatenated value of $d_{j}$ and $X_{j}$ represent partial private key of smart camera *j*
$t_{j}$, $t_{h}$	Time period chosen for smart camera *j* and monitoring device *h*
$C_{j.i}$	Represent the smart camera *j* in cluster *i*
$P_{j}$, $P_{h}$	Part of public values chosen by smart camera and monitoring device
$P_{j_{kgc}}$, $P_{h_{kgc}}$	Part of public values generated by smart camera and monitoring devices using $Pu_{{}_{kgc}}$
$SK_{j}$, $SK_{h}$	Private keys of smart camera and monitoring device
*j*, *h*	Identities of smart cameras and monitoring devices
$d_{j}$, $d_{h}$	partial keys of smart cameras and monitoring devices
$pk_{j}$, $pk_{h}$	Public key of smart camera and monitoring device
$sk_{c_{j.i}}$	Secret keys of smart cameras in cluster *i*
$s_{j}\in Z_{q}^{*}$	Secret value chosen by smart camera during symmetric key generation
$V\in {\left\{0,1\right\}}^{n}$	Value computed by smart camera during signcryption
$K_{enc}$, $K_{dec}$	One-time encryption and decryption keys
*l*	List of the monitoring devices
ϱ(l)	Individual-ciphertext for *h* monitoring devices in *l*
θ	The combined data of individual-ciphertexts
σ	Random number chosen by smart camera *j*
*S*	The sum of the signature part
*f*	Number of frames for the region of interest (RoI)
ϑ	Represent aggregate-signcryptext data
$H_{j}$, $H_{h}$	Hash values generated by smart cameras and monitoring devices
